# Incidence of brain injuries in a large cohort of very preterm and extremely preterm infants at term-equivalent age: results of a single tertiary neonatal care center over 10 years

**DOI:** 10.1007/s00330-024-10592-z

**Published:** 2024-01-27

**Authors:** Karla Drommelschmidt, Thomas Mayrhofer, Britta Hüning, Anja Stein, Borek Foldyna, Bernd Schweiger, Ursula Felderhoff-Müser, Selma Sirin

**Affiliations:** 1 Department of Pediatrics I, Neonatology, Pediatric Intensive Care, and Pediatric Neurology, University Hospital Essen, University of Duisburg-Essen, Essen, Germany; 2 Center for Translational Neuro- and Behavioral Sciences (cTNBS), University Medicine Essen, Essen, Germany; 3 School of Business Studies, Stralsund, University of Applied Sciences, Stralsund, Germany; 4 Cardiovascular Imaging Research Center, Department of Radiology, Massachusetts General Hospital – Harvard Medical School, Boston, MA USA; 5 Department of Diagnostic and Interventional Radiology and Neuroradiology, University Hospital Essen, University of Duisburg-Essen, Essen, Germany; 6 Department of Diagnostic Imaging, University Children’s Hospital Zürich, University of Zürich, Zürich, Switzerland

**Keywords:** Preterm infants, Brain injuries, Magnetic resonance imaging, Brain, Incidence

## Abstract

**Objectives:**

Cerebral magnetic resonance imaging (cMRI) at term-equivalent age (TEA) can detect brain injury (BI) associated with adverse neurological outcomes in preterm infants. This study aimed to assess BI incidences in a large, consecutive cohort of preterm infants born < 32 weeks of gestation, the comparison between very (VPT, ≥ 28 + 0 to < 32 + 0 weeks of gestation) and extremely preterm infants (EPT, < 28 + 0 weeks of gestation) and across weeks of gestation.

**Methods:**

We retrospectively analyzed cMRIs at TEA of VPT and EPT infants born at a large tertiary center (2009–2018). We recorded and compared the incidences of BI, severe BI, intraventricular hemorrhage (IVH), periventricular hemorrhagic infarction (PVHI), cerebellar hemorrhage (CBH), cystic periventricular leukomalacia (cPVL), and punctate white matter lesions (PWML) between VPTs, EPTs, and across weeks of gestation.

**Results:**

We included 507 preterm infants (VPT, 335/507 (66.1%); EPT, 172/507 (33.9%); mean gestational age (GA), 28 + 2 weeks (SD 2 + 2 weeks); male, 52.1%). BIs were found in 48.3% of the preterm infants (severe BI, 12.0%) and increased with decreasing GA. IVH, PVHI, CBH, cPVL, and PWML were seen in 16.8%, 0.8%, 10.5%, 3.4%, and 18.1%, respectively. EPT vs. VPT infants suffered more frequently from BI (59.3% vs. 42.7%, *p* < 0.001), severe BI (18.6% vs. 8.7%, *p* = 0.001), IVH (31.9% vs. 9.0%, *p* < 0.001), and CBH (18.0% vs. 6.6%, *p* < 0.001).

**Conclusion:**

Brain injuries are common cMRI findings among preterm infants with a higher incidence of EPT compared to VPT infants. These results may serve as reference values for clinical management and research.

**Clinical relevance statement:**

Our results with regard to gestational age might provide valuable clinical insights, serving as a key reference for parental advice, structured follow-up planning, and enhancing research and management within the Neonatal Intensive Care Unit.

**Key Points:**

• *Brain injury is a common cMRI finding in preterm infants seen in 48.3% individuals.*

• *Extremely preterm compared to very preterm infants have higher brain injury incidences driven by brain injuries such as intraventricular and cerebellar hemorrhage.*

• *Reference incidence values are crucial for parental advice and structured follow-up planning.*

**Supplementary Information:**

The online version contains supplementary material available at 10.1007/s00330-024-10592-z.

## Introduction

Preterm birth (before < 37 weeks of gestation) is common and affects around 15 million infants worldwide annually (10–12% of all live births, up to 18% in low-income settings [1]). Prematurity complications are the leading cause of mortality among young children (17.7% of children 0–5 years, 36.1% in neonates). Surviving very preterm (VPT, 28 to < 32 weeks of gestation) and extremely preterm (EPT, < 28 + 0 weeks of gestation) infants face an increased risk for major morbidities affecting them till adulthood [2]. Therefore, prematurity results in a major individual and socioeconomic burden [3, 4].

Although the prevalence of severe neurological impairment due to prematurity such as cerebral palsy has declined in recent years, the proportion of VPTs, particularly EPTs with neurologic impairment, has remained high (50%) [4–6]. There is a wide spectrum of neurodevelopmental disabilities including motor, cognitive, and neurosensory deficiencies, as well as emotional and behavioral problems, that are difficult to predict [7, 8]. Due to this high variability of neurological disorders, biomarkers for risk stratification, parental counseling, and therapeutic guidance are urgently needed.

Cerebral MRI (cMRI) at term-equivalent age (TEA) is a promising tool for predicting neurodevelopmental outcomes [2, 9]. CMRI detects precisely subtle changes such as focal or diffuse white matter lesions, low-grade intraventricular hemorrhage (IVH), and small cerebellar hemorrhages (CBH) beyond cranial ultrasound [10–12]. Accurate categorization of brain lesions is essential for risk estimation and identifying high-risk infants. More knowledge about incidences of structural brain lesions is needed to improve prevention, target interventions, and better apply supportive care to optimize functional outcomes in high-risk infants. While management strategies may differ across gestational age (GA) categories, the incidence of individual cMRI findings between VPTs and EPTs needs further exploration.

Current cMRI studies on incidences of brain injuries (BIs) are rare and often derived from small samples or missing hemorrhage-sensitive sequences. In particular, data across different GA is lacking but highly clinically relevant. Therefore, the present study aims to describe the incidence and variety of structural brain abnormalities in a large well-defined cohort of preterm infants born at a large academic center. Moreover, this study aims to describe the incidences in subgroups of VPTs and EPTs across weeks of gestation.

## Methods

### Study design and patient characteristics

We conducted a retrospective observational cohort study assessing clinical and imaging data among all preterm infants born < 32 + 0 weeks of gestation at the level III Neonatal Intensive Care Unit (NICU) of the University Hospital Essen in Germany between 01.01.2009 and 31.12.2018. The Institutional Review Board and Ethics Committee approved the present study, and the need to obtain informed consent was waived due to its retrospective nature. We performed this study according to the Standards of Reporting of Diagnostic Accuracy Studies statement (STARD) [13].

Infants were included in this study if they met the following inclusion criteria: (1) in-house birth and treatment at the University Hospital Essen until discharge (and readmission at TEA) or TEA and (2) survival until TEA. Exclusion criteria were (1) diagnosed or suspected genetic disorders or congenital infections and (2) cMRI at TEA unavailable (lack of parental consent). Infants with genetic disorders and congenital infections (e.g., CMV infections) were excluded, as they can be associated with altered brain structure, with a possible overlap of neurological symptoms.

Clinical data (gestational age, birth weight, birth mode, multiples, sex, percentile, preterm premature rupture of membranes (PPROMs), small for gestational age (SGA, defined as infants with a birthweight < 10th percentile), admission temperature) of the infants with available cMRI were collected and reviewed from the medical reports retrospectively (K.D.).

### Magnetic resonance imaging protocol

MR imaging was performed on a 3-T scanner in most of the infants (*n* = 400, Magnetom Skyra, Siemens Healthcare), if available with a MR-compatible incubator (Lammers Medical Technology (LMT) nomag IC) as previously described [14] or on 1.5-T scanners (*n* = 107, Magnetom Avanto or Magnetom Aera, Siemens Healthcare) (Table 1 suppl.) using a “feed and wrap” technique for immobilization. A more detailed description of the standardized patient preparation is available in our prior publication [14]. Imaging was routinely performed without sedation, and chloral hydrate (20–50 mg/kg) was administered orally if necessary. The routine imaging protocol contained T2-weighted turbo spin echo (TSE) imaging (transversal), a 3D T1-weighted imaging (fast low-angle shot (FLASH), scanned sagittally with transversal and coronal reconstructions), susceptibility-weighted imaging (SWI), and diffusion-weighted imaging (DWI (gradients: b0, b700, b1000)/DTI) as published before [14]. Depending on the underlying pathology, other/additional sequences were performed.

### MRI image analysis

**Fig. 1:**
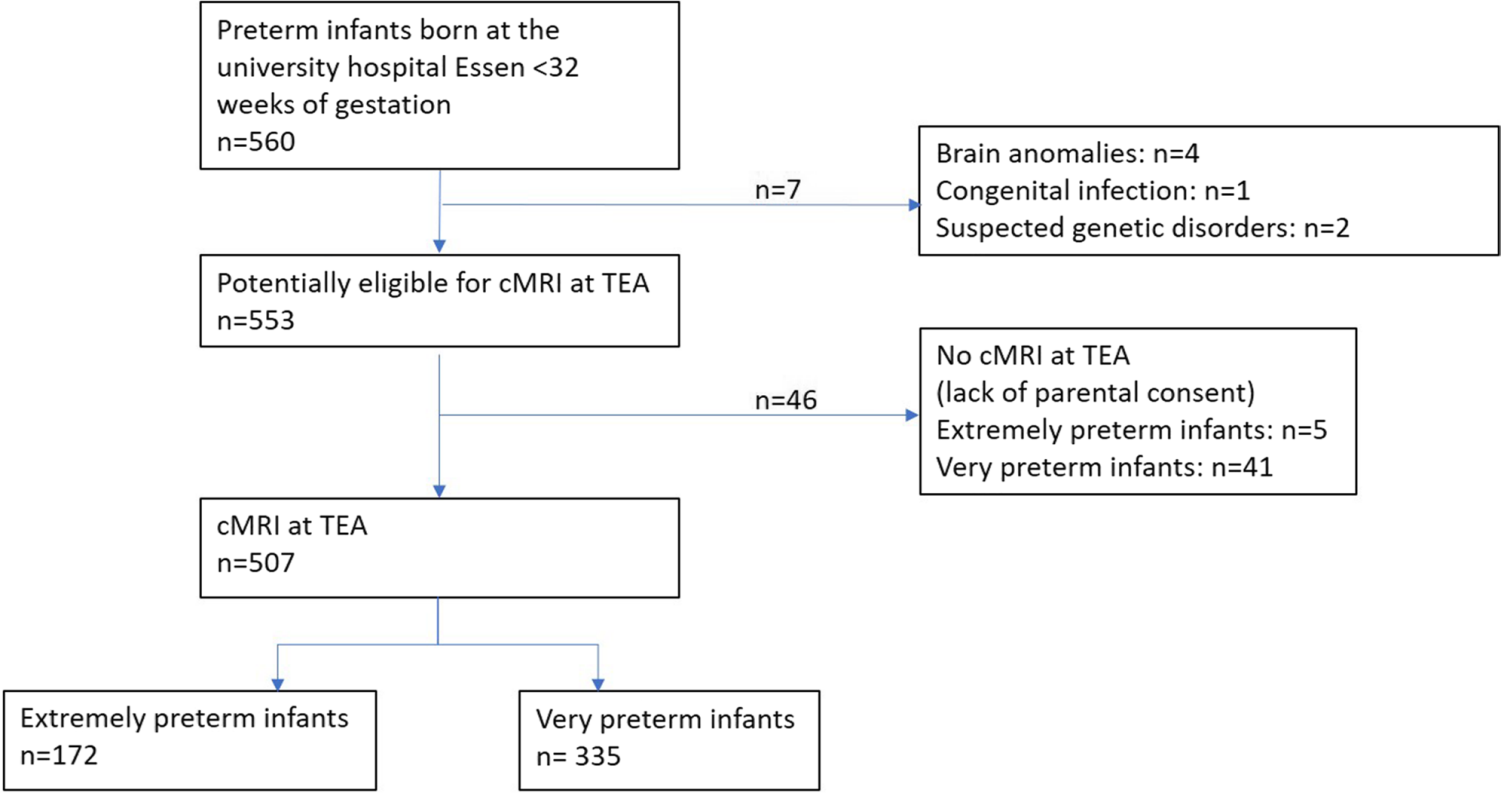
Flowchart of the study population. Extremely preterm infants: infants born < 28 + 0 weeks of gestation; very preterm infants: infants born 28 + 0 to < 32 + 0 weeks of gestation; cMRI at TEA: cerebral MRI at term-equivalent age

Qualitative and quantitative MR image analyses were independently performed by two pediatric radiologists with 14 and 27 years of experience (S.S., B.S.) blinded to clinical data. In case of discrepant results, the final diagnosis was determined by consensus. Interrater reliability was almost perfect for all brain injuries (Cohen’s kappa, 0.81–1; *n* = 38, Table 2 suppl.) [15]. Images were evaluated regarding the presence of IVH I°-III° [16], periventricular hemorrhagic infarction (PVHI) [17], CBH [18], diffuse excessive high signal intensity (DEHSI) [19, 20], ventricular dilatation (VD) [19], punctate white matter lesions (PWML [20, 21], uni-/bilateral and number of lesions/side), and cystic white matter lesions (cPVL, uni-/bilateral) (Fig. 1 suppl.). We defined BI and sBI as follows: BI (IVHI°-III°, PVHI, moderate/severe VD, CBH, PWML, cPVL) and severe BI (sBI) (IVH III°, PVHI, CBH III° + IV°, severe VD, PVL cysts). Presence and number were reported. A detailed description of the classification and grading of preterm brain injuries is given in Table 3 suppl.

### Statistical analysis

Continuous data are presented as mean ± standard deviation (SD) or median and interquartile range (IQR). Categorical and ordinal variables are presented as absolute and relative frequencies. Comparisons were performed using an independent sample *t*-test or Wilcoxon rank-sum test for continuous variables, Fisher’s exact test for categorical variables, and the Wilcoxon rank-sum test for ordinal variables. All statistical analyses (T.M.) were performed using Stata 16.1 (StataCorp LP). A 2-tailed *p*-value < 0.05 was required for all analyses to reject the null hypothesis.

## Results

### Study group

The study population is presented in a flowchart (Fig. 1). Of 650 consecutively born infants in 2009–2018 at our hospital, 560 met the inclusion criteria (77 patients died before TEA, 13 were transferred to other hospitals before TEA), further 53 patients were excluded due to known neurological disease (*n* = 7) or missing cMRI (*n* = 46). MRI scans were performed at TEA at a median of 40 + 1 weeks of gestation (IQR, 0–4) and cMRIs were available in 91.7% (507/553; EPT, 97.2% (172/177); VPT, 89.1% (335/376)). All EPTs from 23, 25, and 26 weeks of gestation were included (100%) and received cMRI (Table 1).

**Table 1:** cMRI at term-equivalent age (eligible and not eligible) according to weeks of gestation

Weeks of gestation	cMRI eligible (*n*/%)	cMRI not eligible (*n*/%)
23	16 (100.0)	0 (0.0)
24	29 (96.7)	1 (3.3)
25	36 (100)	0 (0.0)
26	36 (100)	0 (0.0)
27	55 (93.2)	4 (6.8)
28	60 (98.4)	1 (1.6)
29	78 (89.7)	9 (10.3)
30	94 (87.0)	14 (13.0)
31	103 (85.8)	17 (14.2)

The clinical characteristics of the 507 infants are shown in Table 2. Mean GA was 28 + 2 weeks (SD 2 + 2 weeks, range 23 + 2–31 + 6 weeks), mean birth weight 1170 g ± 387 g (range 420–2200 g) with significantly lower birth weight in the EPTs compared to VPTs (793 g ± 214 g vs. 1364 g ± 303 g, *p* < 0.001). Sex was relatively balanced overall (52.1% boys vs. 47.9% girls) with significantly less boys in the EPTs (43.6% vs. 56.4%, *p* = 0.007). While vaginal delivery rate was lower in the EPTs (2.9% vs. 4.2%, *p* = 0.047), there was no significant difference between the groups regarding multiple births, PPROM, small for gestational age (SGA), and admission temperature.

**Table 2:** Neonatal characteristics of the whole study group, EPT, and VPT infants

Neonatal characteristics (mean ± SD, median (IQR), or *n* (%))	Total (*n* = 507)	EPT infants (*n* = 172)	VPT infants (*n* = 335)	*p*
Weeks of gestation at birth—weeks	28 + 2 (SD 2 + 2)	25 + 4 (SD 1 + 2)	29 + 5 (SD 1 + 2)	< 0.001
Birthweight—g	1.170 ± 386.5	793.1 ± 213.9	1363.9 ± 303.4	< 0.001
Birthweight < 1000 g	182 (35.9)	145 (84.3)	37 (11.0)	< 0.001
Percentile (weight in grams)—%	37.8 ± 23.1	34.1 ± 22.0	39.8 ± 23.4	0.008
Male sex	264 (52.1)	75 (43.6)	189 (56.4)	0.007
Delivery				0.047
Vaginal	19 (3.8)	5 (2.9)	14 (4.2)	
Primary Cesarean section	386 (76.1)	122 (70.9)	264 (78.8)	
Secondary Cesarean section	102 (20.1)	45 (26.2)	57 (17.0)	
Multiple birth	146 (28.8)	45 (26.2)	101 (30.2)	0.407
PPROM—h	0 (0–4)	0 (0–21)	0 (0–2)	0.180
SGA	62 (12.4)	26 (15.3)	36 (10.8)	0.155
Admission temperature—degree Celsius	36.9 ± 0.6	37.0 ± 0.6	36.9 ± 0.6	0.133

*EPT infants* extremely preterm infants born < 28 weeks of gestation, *IQR* interquartile range, *PPROM* preterm premature rupture of the membranes, *SD* standard deviation, *SGA* small for gestational age, *VPT infants* very preterm infants born 28 + 0 to < 32 + 0 weeks of gestation, significant: *p* < 0.05

### cMRI findings at TEA

**Fig. 2:**
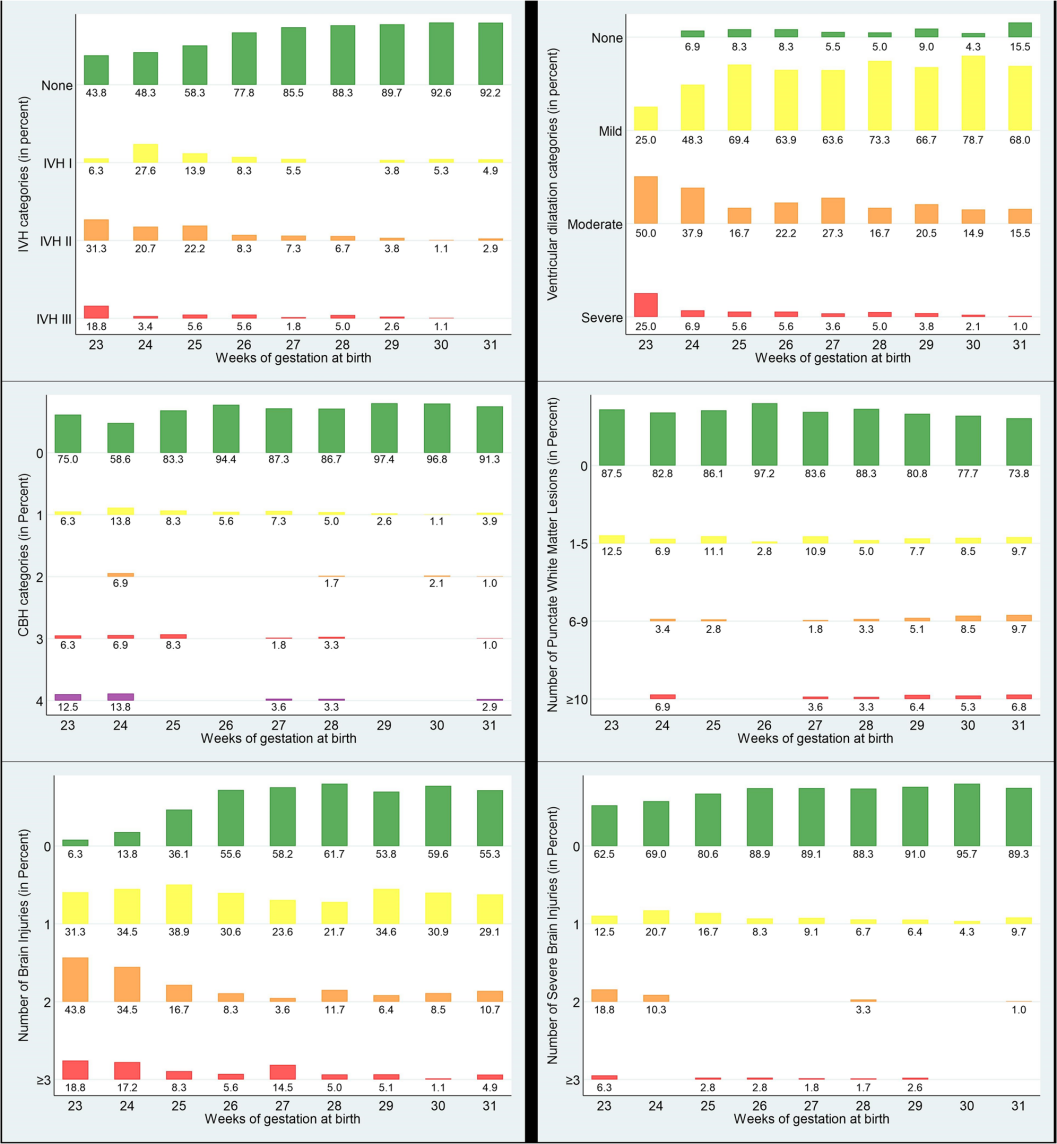
cMRI brain injuries at term-equivalent age stratified by weeks of gestation. *CBH*, cerebellar hemorrhage; *cPVL*, cystic periventricular leukomalacia; *IVH*, intraventricular hemorrhage; *number of brain injuries* (IVH I°-III°, PVHI, moderate and severe VD, CBH, punctate white matter lesions, cPVL); and *number of severe brain injuries* (IVH III°, PVHI, CBH III° + IV°, severe VD, cPVL); *PVHI*, periventricular hemorrhagic infarction; *VD*, ventricular dilatation (mild, moderate, and severe)

**Fig. 3:**
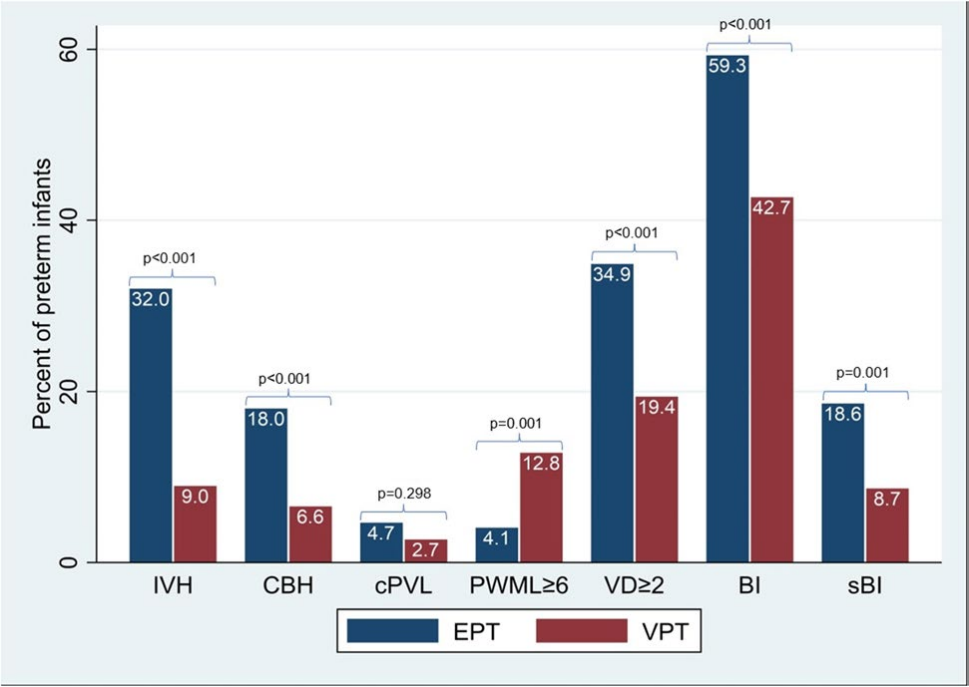
cMRI brain lesions at term-equivalent age stratified by extremely preterm (EPT, < 28 + 0 weeks of gestation) and very preterm (VPT, 28 + 0 to < 32 + 0 weeks of gestation) infants. BI, brain injuries (IVH I°-III°, PVHI, moderate and severe VD, CBH, PWML, cPVL); *CBH*, cerebellar hemorrhage; *cPVL*, cystic periventricular leucomalacia;* IVH*, intraventricular hemorrhage; *PWML*, punctate white matter lesions; *sBI*, severe brain injuries (IVH III°, PVHI, CBH III° + IV°, severe VD, cPVL), *ventricular dilatation* (VD), significant: *p* < 0.05

Diagnostic cMRI was available in all 507 children. The presence of brain injuries according to weeks of gestation is shown in Fig. 2. EPTs showed significantly higher rates of IVH, CBH, and VD compared to VPTs (Table 3/Fig. 2/Fig. 3).

**Table 3:** cMRI at term-equivalent age: Incidence of brain injuries stratified for the study group, EPT (< 28 + 0 weeks of gestation) and VPT (28 + 0 to < 32 + 0 weeks of gestation) infants

Brain injury	Total (*n* = 507) (*n*/%)	EPT infants (*n* = 172) (*n*/%)	VPT infants (*n* = 335) (*n*/%)	*p*
IVH				< 0.001
None	422 (83.2)	117 (68.0)	305 (91.0)	
IVH I°	33 (6.5)	20 (11.6)	13 (3.9)	
IVH II°	37 (7.3)	26 (15.1)	11 (3.3)	
IVH III°	15 (3.0)	9 (5.2)	6 (1.8)	
IVH I °				0.056
Bilateral	9/33 (27.3)	8/20 (40.0)	1/13 (7.7)	
Unilateral	24/33 (72.7)	12/20 (60.0)	12/13 (92.3)	
PVHI	4 (0.8)	2 (1.2)	2 (0.6)	0.607
Ventricular dilatation				0.001
None	41 (8.1)	11 (6.4)	30 (9.0)	
Mild	341 (67.3)	101 (58.7)	240 (71.6)	
Moderate	104 (20.5)	48 (27.9)	56 (16.7)	
Severe	21 (4.1)	12 (7.0)	9 (2.7)	
CBH	53 (10.5)	31 (18.0)	22 (6.6)	< 0.001
CBH Score (Kidokoro)				0.001
None	454 (89.6)	141 (82.0)	313 (93.4)	
CBH I°	24 (4.7)	14 (8.1)	10 (3.0)	
CBH II°	6 (1.2)	2 (1.2)	4 (1.2)	
CBH III°	10 (2.0)	7 (4.1)	3 (0.9)	
CBH IV°	13 (2.6)	8 (4.7)	5 (1.5)	
cPVL				0.478
None	490 (96.7)	164 (95.4)	326 (97.3)	
Bilateral	9 (1.8)	4 (2.3)	5 (1.5)	
Unilateral	8 (1.6)	4 (2.3)	4 (1.2)	
Punctate white matter lesions				0.067
None	415 (81.9)	150 (87.2)	265 (79.1)	
Bilateral	71 (14.0)	16 (9.3)	55 (16.4)	
Unilateral	21 (4.1)	6 (3.5)	15 (4.5)	
≥ 6	50 (9.9)	7 (4.1)	43 (12.8)	0.001
DEHSI	490 (96.7)	169 (98.3)	321 (95.8)	0.196
Number of brain injuries				< 0.001
0	262 (51.7)	70 (40.7)	192 (57.3)	
1	152 (30.0)	53 (30.8)	99 (29.6)	
2	59 (11.6)	28 (16.3)	31 (9.3)	
3	26 (5.1)	16 (9.3)	10 (3.0)	
4	6 (1.2)	3 (1.7)	3 (0.9)	
5	2 (0.4)	2 (1.2)	0 (0.0)	
> 1 Brain injury	93 (18.3)	49 (28.5)	44 (13.1)	< 0.001
Severe brain injuries	61 (12.0)	32 (18.6)	29 (8.7)	0.001
Number of severe brain injuries				0.006
0	446 (88.0)	140 (81.4)	306 (91.3)	
1	45 (8.9)	22 (12.8)	23 (6.9)	
2	9 (1.8)	6 (3.5)	3 (0.9)	
3	5 (1.0)	3 (1.7)	2 (0.6)	
4	1 (0.2)	0 (0.0)	1 (0.3)	
5	1 (0.2)	1 (0.6)	0 (0.0)	
> 1 Severe brain injury	16 (3.2)	10 (5.8)	6 (1.8)	0.028

*Brain injury* (IVHI°-III°, PVHI, moderate and severe VD, CBH, punctate white matter lesions, cPVL); *CBH* cerebellar hemorrhage, *cPVL* cystic periventricular leukomalacia, *DEHSI* diffuse excessive high signal intensity, *EPT* extremely preterm infants born < 28 weeks ofgestation, *IVH* intraventricular hemorrhage, *PVHI* periventricular hemorrhagic infarction; *severe brain injury* (IVH III°, PVHI, CBH III° + IV°, severe VD, cPVL); *VPT* very preterm infants born 28 + 0 to < 32 + 0 weeks of gestation, *VD* ventricular dilatation (mild, moderate, severe); significant: p < 0.05

#### Frequency of brain injury

A total of 245 (48.3%) infants showed BI at cMRI (Table 3), and the incidence decreased with increasing GA (Fig. 2). 93.7% of the infants born at 23 weeks of gestation had at least one, and 62.5% had two or more BIs, whereas 44.7% of the infants born at 31 weeks of gestation had at least one, and 15.5% had two or more BIs. EPTs are significantly more often affected by BI (> 1 BI) and showed a significantly higher number of concomitant BIs compared to VPTs (both *p* < 0.001).

At least one form of sBI was present in 61 (12.0%) infants with higher incidences and numbers of sBI with decreasing weeks of gestation, particularly in infants born at 23 and 24 weeks of gestation (Fig. 2). EPTs suffered significantly more often from sBI compared to VPTs (18.6% vs. 8.7%, *p* = 0.001). At least one form of intracranial hemorrhage (IVH I°-III°, PVHI, CBH) was present in 22.9%, and significantly more often in EPTs compared to VPTs (40.7% vs. 13.7%, *p* < 0.001).

#### Intraventricular hemorrhage

**Fig. 4:**
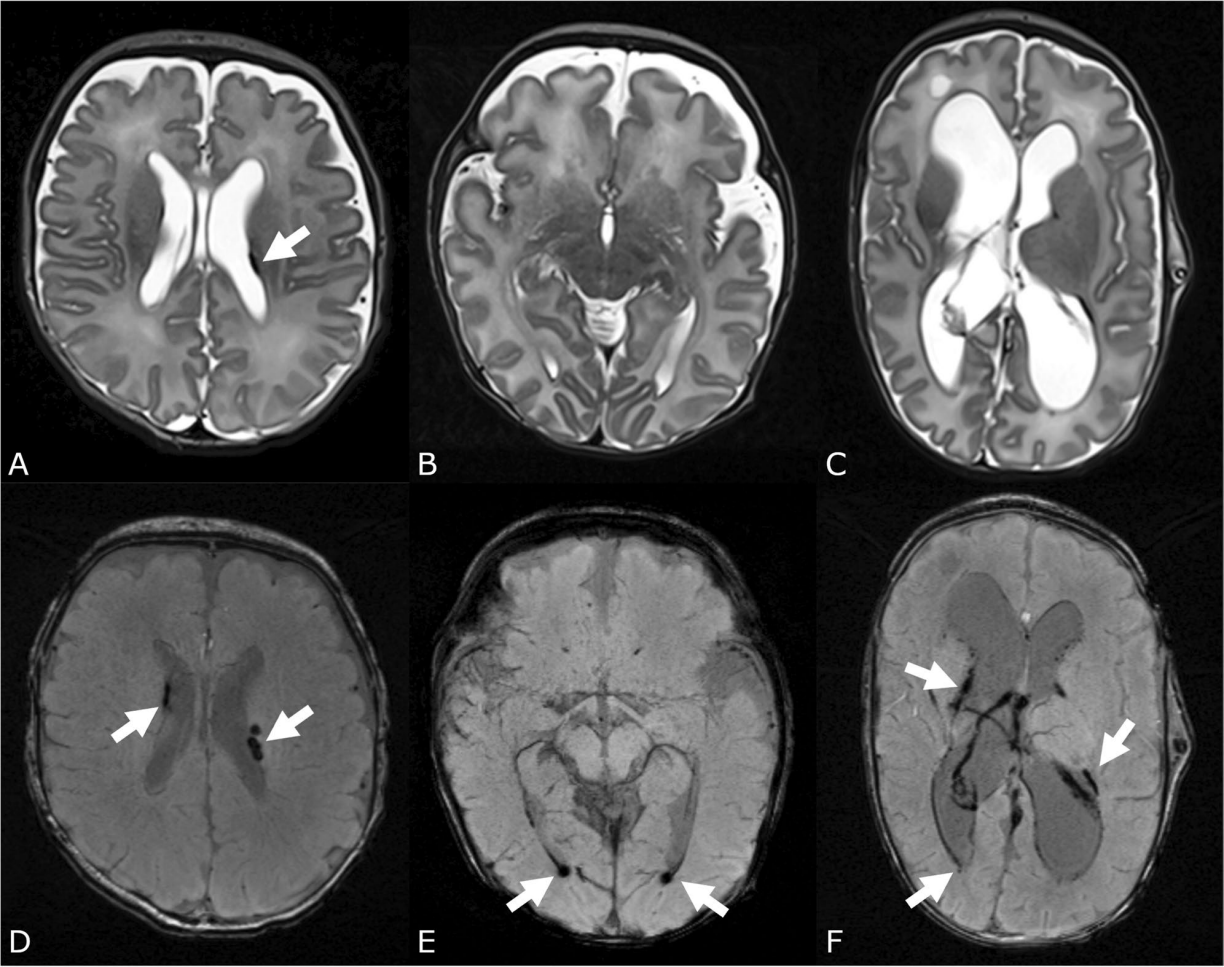
Intraventricular hemorrhage I°-III°. The T2 (**A**) and SWI (**D**) images of a male extremely preterm infant born at 25 weeks of gestation showed IVH I°. IVH I° is visible bilaterally on SWI (**D**), on T2 only on the left side (white arrows). T2 (**B**)- and SWI (**E**)-weighted images of a female very preterm infant (31 weeks of gestation) with IVH II° visible at SWI-weighted image (**E**, white arrows). T2 (**C**) and SWI (**F**) images of a female preterm infant (25 weeks of gestation) with IVH III° (white arrows)

Overall, 16.8% (*n* = 85) of the infants suffered from IVH. The incidence decreased with increasing GA, particularly for IVH II°/III° (Fig. 4). IVH II° decreased from 31.3% at 23 weeks of gestation to 20.7–22.2% at 24–25 weeks of gestation to 8.3–6.7% at 26–28 weeks of gestation to < 5% at 28–32 weeks of gestation. The highest incidence of IVH III° was found in infants born at 23 weeks of gestation (18.8%). It decreased between 24 and 30 weeks of gestation (1.8–5.6%) and was not present in infants born at 31 weeks of gestation. EPTs were significantly more often affected by IVH than VPTs. Incidence of PVHI was rare and did not differ between EPTs and VPTs; concomitant IVH II° or III° was present in all infants.

#### Cerebellar hemorrhage

**Fig. 5:**
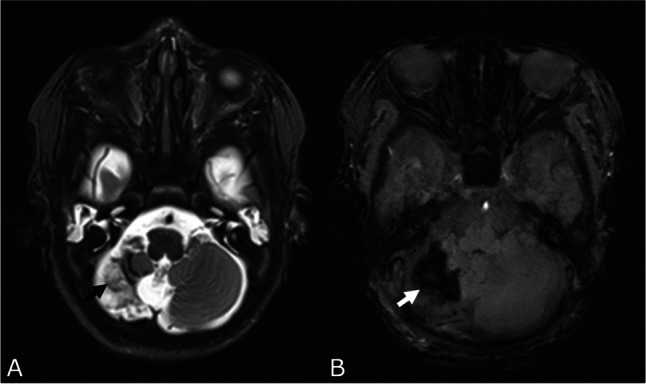
Cerebellar hemorrhage. T2 (**A**) and SWI (**B**) images of a female very preterm infant born at 28 weeks of gestation with a large cerebellar hemorrhage (CBH IV° (Kidokoro), black arrow (**A**) and white arrow (**B**)) and atrophy of the right cerebellar hemisphere

CBH was present in 10.5% (*n* = 53) of the infants with a decreasing incidence with increasing GA at birth (25% in infants born at 23, 8.7% at 31 weeks of gestation). It was also significantly higher in EPTs compared to VPTs (18% and 6.6%, respectively, *p* < 0.001, Fig. 5).

#### Cystic periventricular leucomalacia

**Fig. 6:**
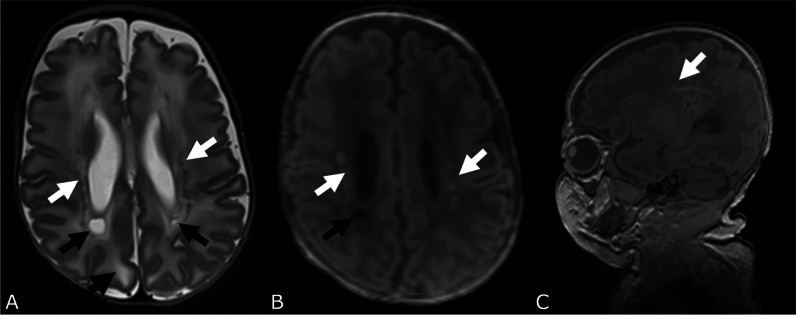
White matter damage. T2 (**A**) and T1 (**B** transverse reconstruction of 3D FLASH, **C** FLASH sequence sagittal) of a female very preterm infant born at 27 weeks of gestation with multiple, bilateral punctate white matter lesions demonstrated as hypointensities (white arrows in **A**) and hyperintensities (white arrows in **B** and **C**), bilateral cystic PVL (black arrows in **A**) and diffuse excessive high signal intensity (DEHSI) (fat black arrow in **A**)

Cystic PVL was present in 3.4% of the infants, with no significant difference between EPTs and VPTs (4.7% and 2.7%, *p* = 0.3, Fig. 6). There was no significant difference in the incidence of unilateral or bilateral cysts between EPTs and VPTs.

#### Punctate white matter lesions

PWML were found in 18.1% (*n* = 92) of the infants with a higher percentage in VPTs compared to EPTs (20.9% and 12.8%, *p* = 0.028). Bilateral PWML were more frequent than unilateral lesions in both groups (Fig. 6). We detected 50 preterm infants with ≥ 6 PWML (VPT 12.8% vs. EPT 4.1%, *p* = 0.001).

#### Diffuse excessive high signal intensity

DEHSI were present in 96.7% (*n* = 490) of the infants, with no significant difference between EPTs and VPTs.

#### Ventricular dilatation

In 91.9% (*n* = 467) of the infants, a VD was present with a decrease in incidence and severity with increasing GA. VD was present in all infants born at 23 weeks of gestation, with a high percentage of high-grade dilatation (severe, 25%), which was rare in infants born at 31 weeks of gestation (1%). A significantly higher average degree of dilatation was present in EPTs compared to VPTs (*p* = 0.001).

## Discussion

We report on brain injury incidences in preterm infants born below 32 weeks of gestation, as determined by cMRI at TEA. Our data covers a near-complete, unselected, and consecutive cohort of preterm infants born at a large tertiary neonatal care center over 10 years. An additional subgroup analysis compared extremely preterm to very preterm infants, and each week of gestation separately. We found that brain injury is a common cMRI finding among preterm infants with a higher incidence in extremely preterm compared to very preterm infants. We also show the distribution of brain injuries across weeks of gestation. These incidences of brain injuries across gestational age can help to improve risk stratification, parental counseling, decision-making, and medical support planning.

Adverse neurological outcome is frequent in preterm infants and ranges from mild cognitive disorders to severe motor deficits like cerebral palsy. In a recent review, the neurological consequences of prematurity and clinical implications were published by Inder et al based on brain injuries detected by cMRI [2], which has been proven to be a promising imaging tool for predicting neurodevelopmental outcomes for many years. In 2006, Woodward et al found that abnormal findings identified by cMRI at TEA in very preterm infants strongly predicted adverse neurodevelopmental outcomes at two years of age [9], which was also confirmed in other studies [10, 18]. As a result, cMRI was implemented in our hospital for all infants born < 32 weeks of gestation as a part of standard clinical practice. Although published cohorts with routine cMRI at TEA already exist, incidences of cMRI findings separately for each week of gestation have, to our knowledge, not been published yet. Additionally, published studies on cohorts with routine cMRI at TEA have been smaller (*n* = 300 [22], *n* = 247 [23]), had more dropouts (84.5% [22]), used only conventional sequences or irregular use of SWI [22, 24], included infants > 32 + 0 weeks of gestation [22, 24], only EPTs [25], or did not differentiate between EPTs and VPTs. Therefore, the presented incidences of BI in preterm infants with regard to GA offer valuable reference data for clinical management and research.

Early identification of high-risk infants for adverse neurological outcomes is essential, as it allows for better obstetrical management and neurorehabilitative support [26], as well as targeted intervention to counteract brain injury [2] throughout the neonatal period as a critical stage of brain plasticity and beyond. It has been shown that the degree of immaturity is the key factor influencing the occurrence of BI. In accordance with previous research, we found an increased incidence of BI with lower GA [5, 27–29]. In our study, infants born < 28 weeks of gestation suffered significantly more frequently from BI, severe BI, IVH, and CBH. Given the vulnerability of this patient group, it is essential to conduct further neonatal research into the variety and frequency of structural brain injuries, in order to improve treatment and outcome.

Intracranial hemorrhage is common in preterm infants and is associated with worse neurological outcomes [2, 23, 30]. As preterm infants often suffer from small or residual hemorrhages, we utilized the highly sensitive SWI sequence to improve hemosiderin detection compared to conventional imaging [31], facilitating an improved classification and detection of hemorrhages. Using SWI may lead to a higher detection rate of small hemorrhages (e.g., punctate hemorrhages, intraventricular blood for differentiation IVH I°/II°) as published before in diverging clinical settings [32, 33]. The incidence of IVH in our study group (16.8%) was in line with published data (14–16.2% [22–24]), as it was for the EPTs separately (32% vs. 32.8% [25]). Incidence of PVHI was low in our group (0.8% vs. 3% [24]). Mostly, incidences of IVH are reported without further differentiation, losing detailed information [18, 22, 34]. In contrast to Buchmayer et al, who also used SWI, we found a lower incidence of IVH I° and a higher percentage of IVH II° in the EPTs (IVH I°, 11.6%/IVH II°, 15.1% versus 14.1%/5.6% [25]), and a lower incidence of IVH III° and PVHI (IVH III°, 5.2%/PVHI, 1.2% versus 8.6%/4.5% [25]). As the impact of IVH I°/II° in opposition to IVH III°/PVHI for the neurodevelopmental outcome is discussed controversially [35, 36] and lacks MRI data [24], we are convinced that a detailed classification should be the basis for further evaluation of the impact of intracranial hemorrhage on neurodevelopmental outcome.

CBH has an substantial, previously underestimated, impact on neurodevelopmental outcomes [2, 37]. Our results corroborate prior studies with an incidence of 10.5% for CBH in infants born < 32 weeks of gestation (10% [18], 8% [22]) and for EPTs (18% vs. 20.7% [25]/19% [38]), even though higher rates of CBHs (30%) are published [24]. In conclusion, CBH is a frequent finding, particularly in EPTs, which may reflect that the third trimester is the critical developmental phase (volumetric growth, 600%) of the cerebellum [39].

White matter damage predicts neurological impairment in preterm infants associated with cognitive and motor delay [2, 9]. CPVL, PWML, and VD are a sign of white matter loss, and DEHSI belong to the preterm-specific injuries and abnormalities of the white matter with multifactorial pathogenesis [40]. CPVL is one of the strongest predictors of adverse neurological outcomes and may result in cerebral palsy, particularly in EPTs [4, 18, 41]. We detected comparable incidences of cPVL with 3.4%, of whom 1.8% were located bilaterally (1.3–3% [24, 42]). Another pathology with presumed association with adverse neurodevelopmental outcome is PWML. It is known that number (> 6) and localization of PWML have an impact on motor, cognitive, and behavioral outcomes of preterm infants [19, 41]. Interestingly, we found a higher incidence in the VPTs compared to the EPTs, which may be caused by the disappearance of these lesions over time until TEA as shown in longitudinal studies [43]. White matter volume loss resulting in VD is supposed to be responsible for neurodevelopmental impairment, particularly motor delay [2, 19]. VD was frequent in our study group ranging from mild to severe degrees. Corroborating previous publications, DEHSI were common in our cohort (96.7%) and we presume these to be temporary prematurity-related brain lesions without pathological impact [19, 44]. Further studies are necessary to discriminate the impact of the different white matter pathologies and to clarify the potential clinical implications of the obtained results.

Our study has several limitations, particularly the retrospective single-center character of our study design. However, the rigorous data collection and curation at our academic center led to only a relatively small number of missing data, particularly in the EPT group, limiting the selection bias and allowing a uniform imaging acquisition (e.g., standardized high-quality sequences, SWI, 3-T scanners) and interpretation by two specialized pediatric radiologists. Additionally, the cross-sectional design of the study does not provide information on the prognostic value of the individual imaging findings. Further, prospective, if possible, multicenter clinical studies are necessary to confirm our results and to determine the prognostic value of imaging findings beyond clinical assessment and conventional imaging.

In conclusion, this study highlights the high prevalence of brain injury in preterm infants, with cMRI revealing more brain injuries in EPT compared to VPT infants and a strong association between lower gestational age and increased brain injury incidences. Knowledge of the frequency of brain injuries, such as intraventricular and cerebellar hemorrhages, is essential for outcome evaluation and is the basis for improving patient management, in particular individually tailored strategies for care in infants born below 32 weeks of gestation.

### Supplementary Information

Below is the link to the electronic supplementary material.

Supplementary file1 (PDF 308 KB)

## Abbreviations

BI: Brain injury
CBH: Cerebellar hemorrhage
cMRI: Cerebral magnetic resonance imaging
cPVL: Cystic periventricular leucomalacia
DEHSI: Diffuse excessive high signal intensity
EPT: Extremely preterm infants
GA: Gestational age
IVH: Intraventricular hemorrhage
PPROM: Preterm premature rupture of membranes
PVHI: Periventricular hemorrhagic infarction
PWML: Punctate white matter lesions
sBI: Severe brain injury
SGA: Small for gestational age
SWI: Susceptibility-weighted imaging
TEA: Term-equivalent age (the regular time point at which the infant would have been born had it not been prematurely delivered)
VD: Ventricular dilatation
VPT: Very preterm infants

## Declarations

### Guarantor

The scientific guarantor of this publication is Selma Sirin, Department of Diagnostic Imaging, University Children’s Hospital Zürich, University of Zürich, Zürich, Switzerland.

### Statistics and biometry

Prof. Thomas Mayrhofer kindly provided statistical advice for this manuscript.

### Study subjects or cohorts overlap

No previously reported subjects or cohorts.

### Methodology

• retrospective

• cross-sectional study

• performed at one institution
