# Supplementary material for: Incidence of brain injuries in a large cohort of very preterm and extremely preterm infants at term-equivalent age: results of a single tertiary neonatal care center over 10 years
Source: Eur Radiol. 2024 Jan 27;34(8):5239–49. doi: 10.1007/s00330-024-10592-z (PMC11255071; doi:10.1007/s00330-024-10592-z)
Supplement: Supplementary file 1 — Supplementary file1 (PDF 308 KB) [file 330_2024_10592_MOESM1_ESM.pdf]

Supplementary material:

Table 1 suppl.: cMRI at term-equivalent age: Incidence of brain injuries stratified by Tesla (1.5 vs. 3 Tesla)

Brain injury	Total (n=507) (n/%)	1.5 Tesla (n=107) (n/%)	3.0 Tesla (n=400) (n/%)	p
IVH				0.371
None	422 (83.2)	85 (79.4)	337 (84.3)	
IVH I°	33 (6.5)	7 (6.5)	26 (6.5)	
IVH II°	37 (7.3)	12 (11.2)	25 (6.3)	
IVH III°	15 (3.0)	3 (2.8)	12 (3.0)	
IVH I °				0.642
Bilateral	9/33 (27.3)	1/7 (14.3)	8/26 (30.8)	
Unilateral	24/33 (72.7)	6/7 (85.7)	18/26 (69.2)	
PVHI	4 (0.8)	1 (0.9)	3 (0.8)	1.000
Ventricular dilatation				0.434
None	41 (8.1)	5 (4.7)	36 (9.0)	
Mild	341 (67.3)	75 (70.1)	266 (66.5)	
Moderate	104 (20.5)	24 (22.4)	80 (20.0)	
Severe	21 (4.1)	3 (2.8)	18 (4.5)	
CBH	53 (10.5)	7 (6.5)	46 (11.5)	0.157
CBH Score (Kidokoro)				0.680
None	454 (89.6)	100 (93.5)	354 (88.5)	
CBH I°	24 (4.7)	3 (2.8)	21 (5.3)	
CBH II°	6 (1.2)	0 (0.0)	6 (1.5)	
CBH III°	10 (2.0)	2 (1.9)	8 (2.0)	
CBH IV°	13 (2.6)	2 (1.9)	11 (2.8)	
cPVL				0.289
None	490 (96.7)	105 (98.1)	385 (96.3)	
Bilateral	9 (1.8)	0 (0.0)	9 (2.3)	
Unilateral	8 (1.6)	2 (1.9)	6 (1.5)	
Punctate white matter lesions				0.387
None	415 (81.9)	83 (77.6)	332 (83.0)	
Bilateral	71 (14.0)	19 (17.8)	52 (13.0)	
Unilateral	21 (4.1)	5 (4.7)	16 (4.0)	
≥6	50 (9.9)	13 (12.2)	37 (9.3)	0.365
DEHSI	490 (96.7)	107 (100.0)	383 (95.8)	0.030
Number of brain injuries				0.635
0	262 (51.7)	57 (53.3)	205 (51.3)	
1	152 (30.0)	28 (26.2)	124 (31.0)	
2	59 (11.6)	12 (11.2)	47 (11.8)	
3	26 (5.1)	9 (8.4)	17 (4.3)	
4	6 (1.2)	1 (0.9)	5 (1.3)	
5	2 (0.4)	0 (0.0)	2 (0.5)	

>1 brain injury	93 (18.3)	22 (20.6)	71 (17.8)	0.486
Severe brain injuries	61 (12.0)	8 (7.5)	53 (13.3)	0.131
Number of severe brain injuries				0.325
0	446 (88.0)	99 (92.5)	347 (86.8)	
1	45 (8.9)	5 (4.7)	40 (10.0)	
2	9 (1.8)	1 (0.9)	8 (2.0)	
3	5 (1.0)	2 (1.9)	3 (0.8)	
4	1 (0.2)	0 (0.0)	1 (0.3)	
5	1 (0.2)	0 (0.0)	1 (0.3)	
>1 Severe brain injury	16 (3.2)	3 (2.8)	13 (3.3)	1.000

Brain injury: IVHI°-III°, PVHI, moderate and severe VD, CBH, punctate white matter lesions, cPVL, *CBH:* cerebellar hemorrhage, *cPVL:* cystic periventricular leukomalacia, *DEHSI:* diffuse excessive high signal intensity, *IVH:* intraventricular hemorrhage, *PVHI:* periventricular hemorrhagic infarction, *severe brain injury:* IVH III°, PVHI, CBH III°+IV°, severe VD, cPVL, *VD:* ventricular dilatation (mild, moderate, severe), significant: $p<0.05$

Table 2 suppl.: Interrater reliability of brain injuries in preterm infants (estimated in a randomly selected subgroup of 38 infants)

**Brain injury**	**Interrater reliability (Cohen´s kappa)**
IVH	1
IVH I°	1
IVH II°	1
IVH III°	1
PVHI	1
VD	1
mild	0.89
moderate	0.81
severe	0.93
CBH	1
Cysts	0.95
Unilateral	0.91
Bilateral	1
Punctate white matter lesions	1
Unilateral	0.84
Bilateral	0.93

CBH: cerebellar hemorrhage, *cPVL:* cystic periventricular leukomalacia, *IVH:* intraventricular hemorrhage, *PVHI:* periventricular hemorrhagic infarction, *VD:* ventricular dilatation (mild, moderate, severe)

Table 3 suppl.: Classification and grading of preterm brain injuries

**Brain injury**	**Classification criteria**	**Classification/reference**
**IVH**	**IVH I°:** hemorrhage limited to germinal matrix<br>**IVH II°:** blood noted in the ventricular system but not distending it<br>**IVH III°:** blood in the ventricles with distension of the ventricles	According to **Papille** et al. 1978 (Papile LA, Burstein J, Burstein R, Koffler H (1978) Incidence and evolution of subependymal and intraventricular hemorrhage: a study of infants with birth weights less than 1,500 gm. J Pediatr 92:529-534) (adapted for MRI)
**PVHI**	region of abnormal signal intensity and/or cystic degeneration adjacent to or communicating with the lateral ventricle, combined with IVH	Adapted to **Volpe** (Volpe JJ (2018) Volpe´s Neurology of the Newborn, 6 edn. Elsevier) (adapted for MRI)
**VD**	**mild** dilatation, but clear concave shape, with modelling to gyri<br>**moderate** VD, borderline shape<br>**severe** VD, rounded ventricular horns, convex shape	Adapted to **Dewan** et al. 2019 (Dewan MV, Herrmann R, Schweiger B et al. (2019) Are Simple Magnetic Resonance Imaging Biomarkers Predictive of Neurodevelopmental Outcome at Two Years in Very Preterm Infants? Neonatology 116:331-340
**CBH**	**CBH I°**: unilateral punctate lesions <-3mm<br>**CBH II°**: bilateral punctate lesions<br>**CBH III°**: unilateral lesions > 3mm<br>**CBH IV°**: extensive lesions bilateral	Adapted to **Kidokoro** et al. 2014 (Kidokoro H, Anderson PJ, Doyle LW, Woodward LJ, Neil JJ, Inder TE (2014) Brain injury and altered brain growth in preterm infants: predictors and prognosis. Pediatrics 134:e444-453)
**cPVL**	**PVL** (4°): cystic lesions in periventricular WM	Adapted to **Kidokoro** et al. 2014 (Kidokoro H, Anderson PJ, Doyle LW, Woodward LJ, Neil JJ, Inder TE (2014) Brain injury and altered brain growth in preterm infants: predictors and prognosis. Pediatrics 134:e444-453)
**PWML**	Focal hyperintensities (T1- weighted images) within the white matter	Adapted to **Martinez-Biarge** et al. 2016 (Martinez-Biarge M, Groenendaal F, Kersbergen KJ et al. (2016) MRI Based Preterm White Matter Injury Classification: The Importance of Sequential Imaging in Determining Severity of Injury. PLoS One 11:e0156245)

**DEHSI**	Increased and/or inhomogenous signal intensity within the white matter (T2-weighted images)	Adapted to **de Bruine** et al. 2011<br>de Bruïne FT, van den Berg-Huysmans AA, Leijser LM et al. (2011) Clinical implications of MR imaging findings in the white matter in very preterm infants: a 2-year follow-up study. Radiology 261:899-906

CBH: cerebellar hemorrhage, *cPVL:* cystic periventricular leukomalacia, *DEHSI:* diffuse excessive high signal intensity, *IVH:* intraventricular hemorrhage, *PVHI:* periventricular hemorrhagic infarction, *PWML:* punctate white matter lesions, *VD:* ventricular dilatation (mild, moderate, severe)

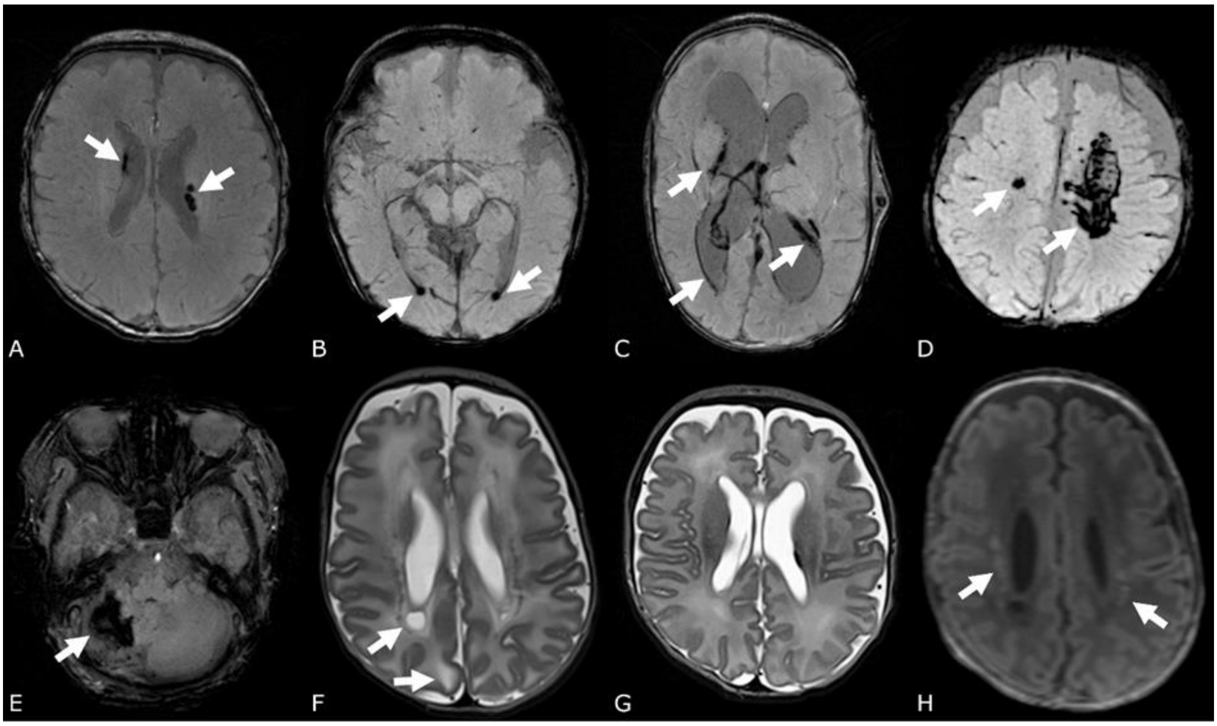

Figure 1 suppl.: Overview of brain injuries in preterm infants. IVH I° (white arrow in A, SWI), IVH II° (white arrow pointing to intraventricular blood in (B), SWI), IVH III° (white arrows pointing to intraventricular blood in (C), SWI), PVHI (white arrows in (D), SWI), CBH IV° (Kidokoro, white arrow in (E), SWI), cPVL (white arrow in (F), T2w), DEHSI (lower white arrow (F), T2w), moderate ventricular dilatation ((G), T2w) and bilateral punctate white matter lesions (white arrows in (H), T1w FLASH)
